# Assumption-checking rather than (just) testing: The importance of visualization and effect size in statistical diagnostics

**DOI:** 10.3758/s13428-023-02072-x

**Published:** 2023-03-03

**Authors:** Itamar Shatz

**Affiliations:** University of Cambridge, Cambridge, UK

**Keywords:** Statistical assumptions, Assumption checks, Statistical diagnostics, Null hypothesis significance testing, Graphical methods, Visualization

## Abstract

Statistical methods generally have assumptions (e.g., normality in linear regression models). Violations of these assumptions can cause various issues, like statistical errors and biased estimates, whose impact can range from inconsequential to critical. Accordingly, it is important to check these assumptions, but this is often done in a flawed way. Here, I first present a prevalent but problematic approach to diagnostics—testing assumptions using null hypothesis significance tests (e.g., the Shapiro–Wilk test of normality). Then, I consolidate and illustrate the issues with this approach, primarily using simulations. These issues include statistical errors (i.e., false positives, especially with large samples, and false negatives, especially with small samples), false binarity, limited descriptiveness, misinterpretation (e.g., of *p*-value as an effect size), and potential testing failure due to unmet test assumptions. Finally, I synthesize the implications of these issues for statistical diagnostics, and provide practical recommendations for improving such diagnostics. Key recommendations include maintaining awareness of the issues with assumption tests (while recognizing they can be useful), using appropriate combinations of diagnostic methods (including visualization and effect sizes) while recognizing their limitations, and distinguishing between *testing* and *checking* assumptions. Additional recommendations include judging assumption violations as a complex spectrum (rather than a simplistic binary), using programmatic tools that increase replicability and decrease researcher degrees of freedom, and sharing the material and rationale involved in the diagnostics.

## Introduction

### Statistical assumptions

Statistical methods, like hypothesis tests and regression models that are often used in the behavioral sciences, generally involve various assumptions. For example, linear models generally involve the assumption that their *residuals*^1^ (i.e., the differences between observed and predicted values) are normally distributed (i.e., have a *Gaussian* distribution). This assumption also applies to common statistical tests that are special cases of linear models, like the *t*-test and ANOVA, as well as to methods that extend these models, like linear mixed-effects models (Barker & Shaw, 2015; Casson & Farmer, 2014; Hox et al., 2018; Knief & Forstmeier, 2021; Pole & Bondy, 2012; Poncet et al., 2016; Rochon et al., 2012; Vallejo et al., 2021; Winter, 2019). Furthermore, it can apply to other quantitative methods, including inferential statistics, like confidence intervals (Alf & Lohr, 2007), and descriptive statistics, like mean and standard deviation (Al-Hoorie & Vitta, 2019). Additional information about this and other assumptions, particularly in the context of linear regression, appears in Appendix 1.

Violations of these assumptions can cause various issues, like statistical errors and biased estimates, whose impact can range from inconsequential to critical (Barker & Shaw, 2015; Ernst & Albers, 2017; Gel et al., 2005; Hayes & Cai, 2007; Hu & Plonsky, 2021; Knief & Forstmeier, 2021; Poncet et al., 2016; Rosopa et al., 2013; Schmidt & Finan, 2018; Troncoso Skidmore & Thompson, 2013; Vallejo et al., 2021; Zuur et al., 2010). Accordingly, it is recommended to consider the assumptions of statistical methods when using those methods, and to use statistical diagnostics to determine whether any assumptions are violated, and if so then how they are violated and to what degree (Barker & Shaw, 2015; Casson & Farmer, 2014; Gel et al., 2005; Hox et al., 2018; Osborne & Waters, 2003; Poncet et al., 2016; Schmidt & Finan, 2018; Tay et al., 2016; Zuur et al., 2010). When violations are detected, the diagnostics can also drive the decision of what to do; common options include switching methods (e.g., to robust non-parametric ones), transforming the data (e.g., by taking its logarithm), or sticking to the original analysis (Casson & Farmer, 2014; Pek et al., 2018; Pole & Bondy, 2012; Vallejo et al., 2021).

### Motivation for this paper

As discussed above, checking assumptions is crucial to ensuring the validity of statistical analyses.

However, the way assumptions are currently checked is often flawed, due to issues like the use of statistical tests in a way that is likely to involve false positives, and these issues persist despite having been mentioned in various previous works (Anderson et al., 2001; Bilon, 2021; Cumming, 2014; Di Leo & Sardanelli, 2020; Ernst & Albers, 2017; Gelman & Stern, 2006; Knief & Forstmeier, 2021; Kozak & Piepho, 2018; Lakens, 2021; Rosnow & Rosenthal, 1989; Tijmstra, 2018; Wasserstein & Lazar, 2016; Winter, 2019; Zuur et al., 2010). Furthermore, lack of awareness and understanding of these issues contributes to the currently insufficient use and reporting of assumption checks in the scientific literature (Hoekstra et al., 2012; Hu & Plonsky, 2021; Nielsen et al., 2019; Nimon, 2012).^2^ For example, a review and empirical analysis by Hu and Plonsky (2021) suggest that assumption checks are likely reported in under 25% of studies in social-science fields like linguistics, psychology, and education, and that many of these reports are lacking (e.g., because they mention only some of the relevant checks). This is supported by other research in the social sciences, such as a study by Ernst and Albers (2017), who found that in psychology research involving linear regression, only 2% of studies were both transparent and correct in reporting assumption checking, and a further 6% were transparent but incorrect. This is also supported by research in other fields, like medicine (e.g., Nielsen et al., 2019), though more research is needed in order to determine the exact rate of reporting, especially to understand how it varies across fields and whether it is increasing over time (Hu & Plonsky, 2021).

One reason for the persistence of the issues with assumption checks is insufficient statistical literacy among researchers, so a possible partial solution is to develop relevant resources on proper assumption-checking, which researchers can learn from (Hu & Plonsky, 2021; Loewen et al., 2014). There are already, as noted above, many works that mentioned these issues. However, they are generally limited, in the sense that they either do so only briefly and in passing (e.g., Winter, 2019), focus on only one or some of these issues (e.g., Tijmstra, 2018), and/or discuss these issues outside the context of statistical diagnostics (e.g., Gelman & Stern, 2006). Furthermore, some works (e.g., Bilon, 2021) present these issues from a technical perspective (e.g., using equations), which readers may struggle to understand and translate into practice, especially if they lack a strong quantitative background, as is often the case (Hu & Plonsky, 2021). All this is *not* meant to criticize these works, which simply had a different focus (e.g., exploring a single issue), but is rather meant to point out an existing and important gap in the literature.

The goal of the present paper is to address this gap, and to consequently improve the way assumption checking is conducted. Specifically, the paper expands on previous work in several ways. First, it aggregates the key common issues with assumption checking, to discuss all of them in one place. Furthermore, it illustrates these issues in a manner that is meant to be intuitive and non-technical, in order to make the material accessible to diverse audiences, including those who have only limited statistics expertise but nevertheless use statistical methods in their work (Hu & Plonsky, 2021). Finally, it takes advantage of the aforementioned aggregation of these issues, in order to synthesize generalizable practical recommendations for improving assumption checking, which again should be accessible to diverse audiences.

The present paper therefore aims to serve as a resource that can be used in several key ways. First, it can be used by researchers to learn how to conduct better statistical diagnostics, and also to explain the rationale behind their diagnostic approach to readers and reviewers, by serving as a comprehensive reference. In addition, this paper may also be used by reviewers and editors, who can use it to guide the statistical diagnostics of authors, by mentioning it during the review process, and potentially also listing it as a methodological resource in the submission guidelines of journals (Hu & Plonsky, 2021; Loewen et al., 2014). Finally, it can also be used for pedagogical purposes, for example by educators who wish to direct their students to an accessible paper that explains how to conduct statistical diagnostics. This aligns with calls to improve the current state of statistical diagnostics in research (Hu & Plonsky, 2021; Nielsen et al., 2019; Nimon, 2012). This also aligns with the goal of *Behavior Research Methods* (BRM) to publish, among other things, “tutorials alerting readers to avoidable mistakes that are made time and time again” (Brysbaert et al., 2020, p. 1), in order to make research “more effective, less error-prone, and easier to run” (ibid.).

## Brief overview of assumption testing

It is often recommended to *test* the assumptions of statistical methods before using them, using *null hypothesis significance tests* (NHST, sometimes referred to in this context as *numerical tests*). For example, when assessing the normality of residuals, a common recommendation is to use the *Shapiro–Wilk test* (Gel et al., 2005; Ghasemi & Zahediasl, 2012; Knief & Forstmeier, 2021; Mishra et al., 2019; Rochon et al., 2012). Generally, when this approach is used, if the resulting *p*-value of the test is < .05, then the residuals are considered significantly non-normal (i.e., the null hypothesis that the data is normally distributed is rejected), meaning that the assumption of normality is considered to be violated.

This approach to checking assumptions can be appealing for various reasons. For example, it involves a single well-established threshold (generally *p* < .05), which reduces some of the arbitrariness and researcher degrees of freedom when using such checks (Wicherts et al., 2016).^3^ Second, it relies on the NHST framework, which many researchers are familiar with and are already using extensively in other parts of their work (Tijmstra, 2018; Troncoso Skidmore & Thompson, 2013; Veldkamp, 2017). Finally, it involves tests that are generally easy to implement from a programmatic perspective, and that are often reported automatically by certain software, so researchers may have the results of these tests available to them by default (Hoekstra et al., 2012).

However, as will be shown in the next section, there are various issues with this approach, which can cause serious problems for those who use it.

## Issues with testing assumptions

The following subsections illustrate the key issues with using assumption tests for diagnostics of statistical methods. These issues include statistical errors, false binarity, limited descriptiveness, misinterpretation, and potential testing failure due to unmet test assumptions.

### Statistical errors

Testing assumptions can cause both *false positives* (i.e., *type I errors*) and *false negatives* (i.e., *type II errors*), as shown below.

False positives occur when a test incorrectly leads to the conclusion that there is an assumption violation, in cases where there is no such violation. For example, this can happen if the test incorrectly leads to the conclusion that a certain distribution is non-normal, in a situation where it is actually normal (and should be normal). This issue is especially common with large samples, where even tiny, random, and inconsequential deviations from an expected distribution often lead to statistically significant differences from that distribution (Bilon, 2021; Bishara et al., 2021; Kozak & Piepho, 2018; Mishra et al., 2019).

**Fig. 1:**
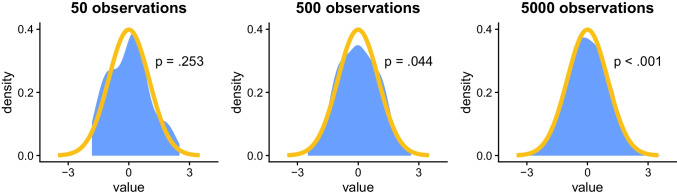
**General background**: The *x*-axis indicates standardized values (*mean* = 0, s*tandard deviation* = 1); the *y*-axis indicates value density (e.g., 0.2 means 20% of observations have this value). The orange line indicates the expected density for a normal distribution; the blue shaded area indicates the observed density for the samples. *p*-values are from Shapiro–Wilk normality tests. **Specific background**: Each sample was randomly generated using identical settings to have a roughly normal distribution with random noise. Samples differ in size, which increases from 50 → 500 → 5000 (left-to-right). Note that the leftmost panel is not truncated, but because it has fewer observations, by chance none are more than 1.8 SD below the mean. **Takeaway**: As sample size increases, the observed distribution approaches normality but the *p*-value decreases, illustrating the risk of false positives in assumption testing, especially in large samples

This issue is illustrated in Fig. 1. The plots it contains show that, as the sample size increases (going from left to right), the distribution of the randomly generated samples approaches normality, as indicated by the observed distribution (the blue shaded area) aligning with an expected normal distribution (the orange line). However, these plots also show that as the sample size increases—and the sample approaches normality—the *p*-value of the associated assumption test decreases. Paradoxically, this means that the smallest sample (*N* = 50), which is the least normal, might be interpreted as the only sample where the normality assumption is not violated (since *p* > .05). Conversely, the medium sample (*N* = 500), which is closer to normality, might be interpreted as non-normal (but somewhat borderline, since *p* = .044), and the largest sample (*N* = 5000), which is closest to normality, might be interpreted as entirely non-normal (since *p* < .001).

This figure demonstrates that, although increasing the sample size is generally beneficial to statistical analyses, it can cause issues when testing assumptions, since large samples are likely to appear to be significantly different from expected distributions according to NHST (Bilon, 2021; Bishara et al., 2021;Kozak & Piepho, 2018 ; Mishra et al., 2019). This *size-significance paradox* of assumption testing can lead to unwarranted lack of confidence in results from large and “overpowered” samples, where minor assumption violations may be incorrectly interpreted as worse than they are (Bishara et al., 2021; Kozak & Piepho, 2018).

Conversely, the second type of statistical errors that assumption tests can cause—false negatives—occur when a test incorrectly leads to the conclusion that there is no assumption violation, in cases where there is one. For example, this can happen if the test leads to the conclusion that a certain distribution is normal (or more accurately, not significantly non-normal), in a situation where it is not. This issue is especially common with small samples, where even substantial and systematic deviations from an expected distribution may not be statistically significant, due to insufficient statistical power (i.e., insufficient ability to detect such deviations at a statistically significant level) (Kozak & Piepho, 2018; Mishra et al., 2019).

**Fig. 2:**
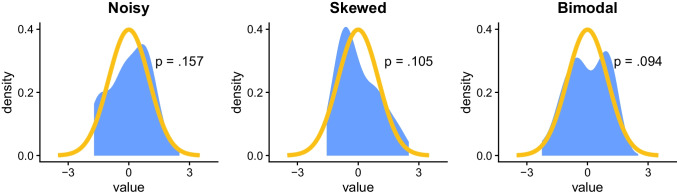
**General background**: The *x*-axis indicates standardized values (*mean* = 0, s*tandard deviation* = 1); the *y*-axis indicates value density (e.g., 0.2 means 20% of observations have this value). The orange line indicates the expected density for a normal distribution; the blue shaded area indicates the observed density for the samples. *p*-values are from Shapiro–Wilk normality tests. **Specific background**: The first sample (left-to-right) is normally distributed but noisy, the second is skewed, and the third is bimodal (*N* = 30 in all samples). **Takeaway**: Despite the substantial deviations from normality, the tests do not detect non-normality, illustrating the risk of false negatives in assumption testing, especially in small samples

This issue is illustrated in Fig. 2. The plots that it contains show three samples with substantial deviations from normality—due to noise, skewness, and bimodality—as indicated by the shape of the observed distributions (blue shaded area). However, in all these cases, the samples might be interpreted as normal based on the associated assumption test (since *p* > .05).

Accordingly, assumption testing can also lead to unwarranted confidence in small and underpowered samples, where the tests are sometimes unable to detect even strong assumption violations.^4^ Together with the issue of false positives, this ironically means that, according to assumption tests, large and normal sample can sometimes be seen as non-normal, whereas small and non-normal samples can sometimes be seen as normal.

### False binarity

**Fig. 3:**
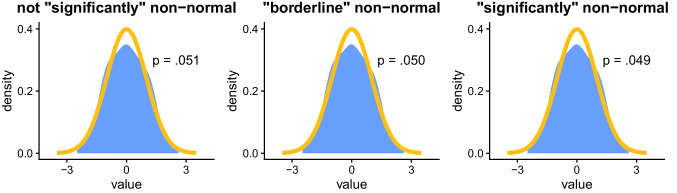
**General background**: The *x*-axis indicates standardized values (*mean* = 0, s*tandard deviation* = 1); the *y*-axis indicates value density (e.g., 0.2 means 20% of observations have this value). The orange line indicates the expected density for a normal distribution; the blue shaded area indicates the observed density for the samples. *p*-values are from Shapiro–Wilk normality tests. **Specific background**: Each sample (*N* = 500) was randomly generated to have a normal distribution with slightly more noise than the previous (left-to-right). **Takeaway**: Based on assumption tests with a hard threshold (*p* < .05), the normality of these samples is classified differently, even though the differences in normality between the samples are tiny

Assumption tests generally involve a hard *threshold* (or *cutoff*), so assumption violations are determined only based on whether *p* < .05, in a *binary* (or *dichotomous*) way. This can lead to completely different interpretations of the data based on inconsequential differences in *p*-values (Gelman & Stern, 2006; Greenland et al., 2016; Halsey, 2019; Wasserstein & Lazar, 2016). For example, as shown in Fig. 3, if the result of a normality test is *p* = .051, then the sample might be considered “normal” (or more accurately, not “significantly” non-normal), whereas if the result is *p* = .049, then the sample might be considered “significantly” non-normal, even though the difference between these values is functionally meaningless.^5^

**Fig. 4:**
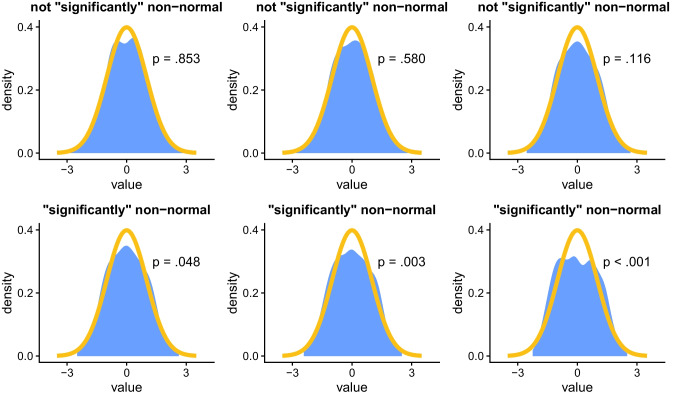
**General background**: The *x*-axis indicates standardized values (*mean* = 0, s*tandard deviation* = 1); the *y*-axis indicates value density (e.g., 0.2 means 20% of observations have this value). The orange line indicates the expected density for a normal distribution; the blue shaded area indicates the observed density for the samples. *p*-values are from Shapiro–Wilk normality tests. **Specific background**: Each sample (*N* = 500) was randomly generated to have a normal distribution, with substantially more noise going from left-to-right and then top-to-bottom. **Takeaway**: Assumption tests with a hard threshold (*p* < .05) designate the top plots as “normal” (or more accurately, as not non-normal) and the bottom plots as non-normal, but do not capture substantial differences in distributions within each group (e.g., the increased non-normality in the bottom-right plot compared to the bottom-left one)

In addition, this binary thinking also compresses a diverse spectrum of possible assumption violations into a narrow false dichotomy. This simplistic view of assumption violations ignores potential nuances, such as that there are different *types* of violations (as was shown in Fig. 2 and will be shown in the next subsection), as well as different *magnitudes* of violations. This issue with magnitude is illustrated in Fig. 4, where, for example, the bottom-right plot appears substantially more non-normal than the bottom-left plot, but both may simply be considered as “non-normal” based on an assumption test (since *p* < .05 in both cases). Note that this plot also illustrates an associated issue with using a hard threshold in assumption tests, since the bottom-left and bottom-right plots are both categorized as non-normal, even though the distribution of the bottom-left plot is more similar to that of the top-right plot (which is not non-normal).

### Limited descriptiveness

**Fig. 5:**
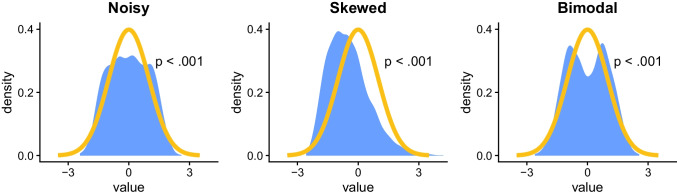
**General background**: The *x*-axis indicates standardized values (*mean* = 0, s*tandard deviation* = 1); the *y*-axis indicates value density (e.g., 0.2 means 20% of observations have this value). The orange line indicates the expected density for a normal distribution; the blue shaded area indicates the observed density for the samples. *p*-values are from Shapiro–Wilk normality tests. **Specific background**: The first sample (left-to-right) is normally distributed but noisy, the second sample is skewed, and the third is bimodal (*N* = 3000 in all samples). **Takeaway**: The assumption tests indicate that there is non-normality in all three samples, but do not reveal the substantially different ways in which the samples are non-normal

Assumption tests, particularly when used with a binary mindset, generally only indicate whether the distribution at hand is “significantly” different from some expected distribution (Greenland et al., 2016; Wasserstein & Lazar, 2016). However, this does not indicate much about how different the distribution is from expected (in terms of magnitude), as was shown in Fig. 4, or in what way the distribution is different, as was shown in Fig. 2. This latter issue is further illustrated in Fig. 5, which contains three plots, each representing a sample that deviates significantly from normality (due to noise, skewness, and bimodality, as indicated by the shape of the observed distributions). Here, the assumption tests detect the assumption violation (unlike in Fig. 2, where they failed to do so), but do not provide any further information about its nature, since all tests merely indicates that *p* < .001.

The informativeness of graphical methods compared to numerical methods is also illustrated in *Anscombe's quartet* (Anscombe, 1973) and the *Datasaurus dozen* (Matejka & Fitzmaurice, 2017), which appear in Appendix 2. These are collections which show how data with very different distributions can have the same summary statistics (e.g., mean, SD, and correlation).

### Misinterpretation

The results of assumption tests can be misinterpreted due to issues that commonly occur when people interpret the results of NHST (Gelman & Stern, 2006; Greenland et al., 2016; Lakens, 2021; Wasserstein & Lazar, 2016). For example, the result of tests might be misinterpreted as suggesting that there is a substantial difference between *p* = .051 and *p* = .049 in the Shapiro–Wilk test, even though this difference is generally meaningless (Gelman & Stern, 2006; Wasserstein & Lazar, 2016), as was shown in the previous sub-section on false binarity. Similarly, the resulting *p*-value might be incorrectly interpreted as an effect size, for instance if people assume that a low *p*-value from the Shapiro–Wilk test (e.g., *p* < .001) indicates strong non-normality (Wasserstein & Lazar, 2016). In addition, non-significant results might be misinterpreted as indicating that a sample is “significantly” normal, even though the associated test only shows that we cannot reject the null hypothesis that the distribution is normal (Greenland et al., 2016; Wasserstein & Lazar, 2016).

### Potential testing failure due to unmet test assumptions

In addition to the aforementioned issues with assumptions tests, which pertain primarily to statistical significance, another issue is that the tests themselves can have various assumptions. This adds further complexity to the diagnostics, as well as more room for error, since researchers can neglect to account for these added assumptions.

**Fig. 6:**
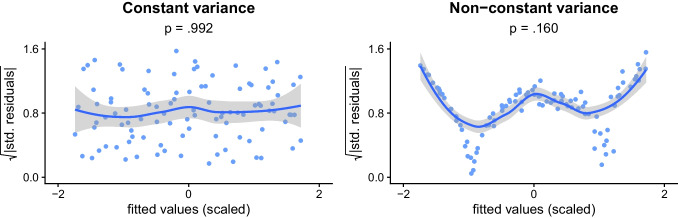
**Background**: Each scatterplot shows the square root of the absolute values of standardized residuals in a simple regression model (*N* = 100), as a function of their scaled fitted values. Each plot also contains a corresponding smoothed regression line, with a grey band for the 95% CI. Constant variance is indicated by a flat and horizontal line, with residuals spread randomly around it. *p*-values are from *Breusch–Pagan tests*. **Takeaway**: There is clear non-constant variance in the right plot, since the line is heavily curved and the residuals are systematically distributed around it, but the associated assumption test fails to detect this

To illustrate this, we will look at another common assumption; that a model’s residuals have *constant variance* (Casson & Farmer, 2014; Cook & Weisberg, 1983; Hayes & Cai, 2007; Rosopa et al., 2013; Schmidt & Finan, 2018; Zuur et al., 2010). As with the normality assumption, testing this assumption can also lead to various issues, like false negatives. This is illustrated by the right plot in Fig. 6, where the test fails to detect a clear pattern of non-constant variance, which is indicated by the curved shape of the line and systematic—rather than random—distribution of the residuals around it.

**Fig. 7:**
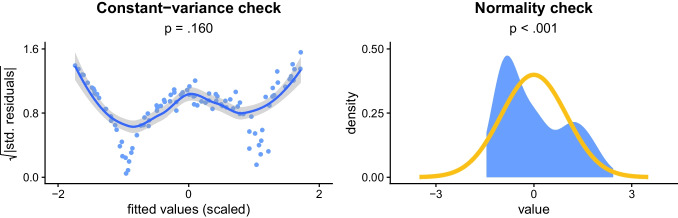
**Background**: The left plot shows a constant-variance check, for the model with non-constant variance shown in Fig. 6. The right plot shows a normality check for this model (based on the paradigm described in Figs. 1, 2, 3, 4 and 5). **Takeaway**: The violation of the normality assumption hinders the ability of the Breusch–Pagan test to detect the non-constant variance

A potential reason for this issue, beyond sample size, is that the commonly used *Breusch–Pagan test* of constant variance itself has assumptions, including normality of residuals, violations of which can cause failure to detect non-constant variance (Barker & Shaw, 2015; Cribari-Neto & Zarkos, 1999; Halunga et al., 2017; Waldman, 1983). This is illustrated in Fig. 7, where, despite clear visual patterns of non-constant variance (as indicated by the regression line being curved and having the residuals distributed systematically around it), the associated Breusch–Pagan test fails to detect the violation, partially due to the violation of the normality assumption.

## A path forward

In the previous section, we saw the key issues associated with assumption testing. In this section, we will see practical recommendations for improving statistical diagnostics, especially in light of the aforementioned issues.

### Beware the issues with assumption testing

One way to minimize the issues that we saw with assumption tests is to account for these issues when using such tests. For example, to avoid false binarity when interpreting the results of a Shapiro–Wilk test, remember that the *p*-value is measured on a continuous spectrum (e.g., there is little difference between *p* = .051 and *p* = .049). In addition, to minimize these issues, you should consider them when deciding whether to use assumption tests in the first place, as there are cases where it is preferable to replace or supplement them with alternative checks, as discussed next.

### Use visualizations in statistical diagnostics

When seeing the issues with assumption tests, we also saw how graphical methods, like residual plots, can help reduce or avoid these issues, and consequently improve statistical diagnostics. This means that you will often benefit from using graphical methods in your diagnostics, as has also been noted by others (Bilon, 2021; Hox et al., 2018; Knief & Forstmeier, 2021; Kozak & Piepho, 2018; Winter, 2019; Zuur et al., 2010).

**Fig. 8:**
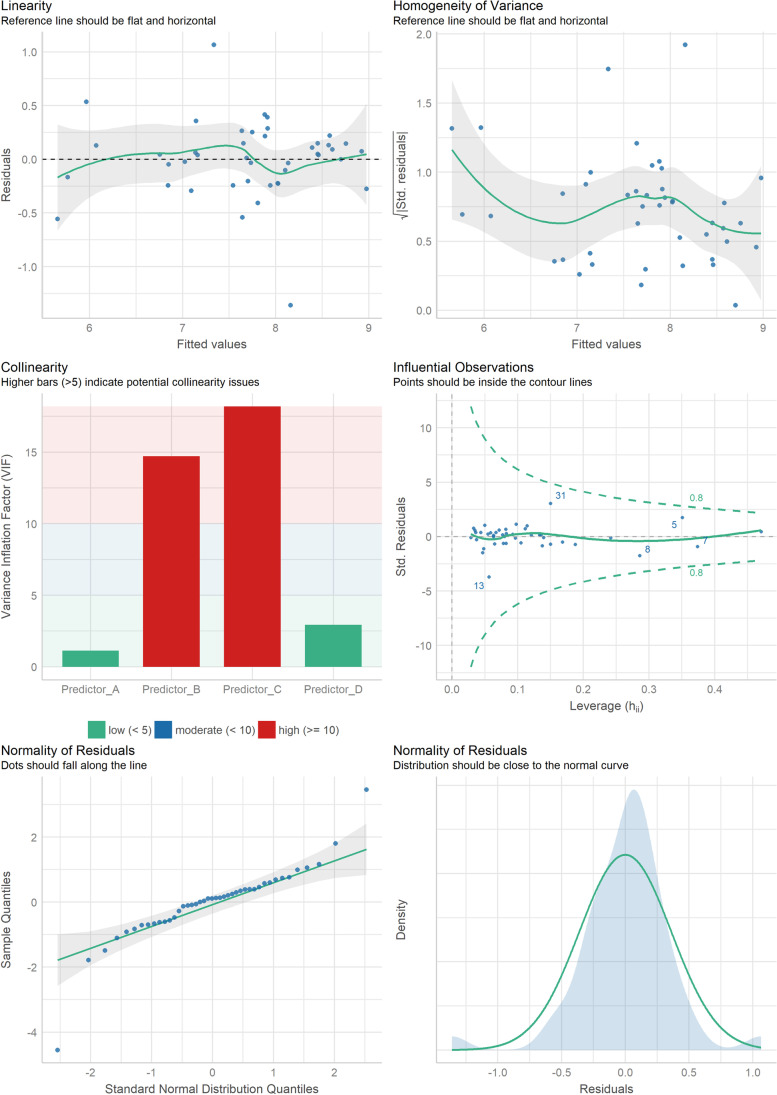
Diagnostics for a regression model, generated using the *performance* package in R (Lüdecke et al., 2021), by simply running *check_model*(model_name). These checks are for linearity, homogeneity of variance (i.e., constant variance), collinearity, influential observations, and normality of residuals; for more details, see the package’s documentation

In addition, using such diagnostics is becoming easier than ever, and in many cases is as easy as using assumption tests. This is illustrated in Fig. 8, which shows how a range of relevant visual assumption checks can be generated with a simple function in R.^6^

When using graphical methods, there is the question of how to conduct *graphical inference* (or *visual statistical inference*), which involves drawing conclusions about statistical properties using visualizations (Hullman & Gelman, 2021; Loy, 2021; Majumder et al., 2013; Wickham et al., 2010). In the present context, a key question is how to judge the severity of assumption violations, in order to determine whether a certain visual pattern deviates enough from expected to merit a response. Though the question of graphical inference is under active consideration (Hullman & Gelman, 2021), and though there is no perfect method for this, there are nevertheless some methods that can help.

One such method is to use visual aids, like confidence intervals that are overlain on plots, to add information regarding the certainty associated with the visual patterns. Examples of this were shown in Fig. 8 (e.g., in the homogeneity of variance plot), where they were generated automatically by the *performance* package in R. A similar approach is utilized in the R *qqtest* package, which generates *self-calibrating* QQ-plots that visually incorporate sampling variation into the display (Oldford, 2016). Furthermore, it is sometimes beneficial to supplement visualizations with numerical aids, including effect sizes and statistical significance (as will be discussed in the next two sub-sections), since they may provide complementary information that aids the judgment and decision-making process (Flatt & Jacobs, 2019; Hartig, 2021).

Another method you can use is the *lineup protocol*, (Buja et al., 2009; Loy, 2021; Majumder et al., 2013; Wickham et al., 2010). This involves generating (e.g., using simulation) a random set of similar distributions that do *not* contain the assumption violation of interest (e.g., residual non-normality), and then checking if you and others can identify the plot containing the original data out of the ones containing the data without a violation. The less able people are to identify the original plot, the less likely it is that it involves a substantial violation (Buja et al., 2009; Loy, 2021; Majumder et al., 2013; Wickham et al., 2010). This protocol—as well as the Rorschach protocol which is mentioned next—can be implemented using programmatic tools like the *nullabor* package in R (Wickham et al., 2010).

In addition, to better understand how to judge visual patterns, it could help to look at relevant examples that are used to illustrate the presence/absence of violations, for example in package documentation (e.g., of the R *DHARMa* package, which is discussed in the next sub-section), or in other instructional sources (e.g., Winter, 2019). When doing this, you can also use the *Rorschach protocol*, by examining randomly generated plots in which assumptions are not violated (e.g., without non-normality). This can help calibrate your expectations regarding the variability that such plots can involve, to reduce the tendency to view random patterns in the data as assumption violations (Buja et al., 2009; Wickham et al., 2010).

Furthermore, when assessing visual patterns, you can ask for input from other individuals (Majumder et al., 2013). In doing so, you should prioritize input from those with expertise in interpreting such plots, who may be able to judge them better, and try to not reveal the goals of the research or any previous judgments of the visual patterns until after these individuals provided their input, to minimize potential bias (Veldkamp, 2017; Wicherts et al., 2016).

Finally, once you make a decision regarding a visual assumption check, you should describe your rationale, for example by explaining which visual patterns you found concerning and why. You should also share the relevant plots (as supplementary material if necessary), in order to be transparent and ensure that other researchers can see these visualizations and apply their own judgment to them.

### Use effect sizes in statistical diagnostics

There are situations where you can benefit from using numerical measures of effect size in your diagnostics, to help identify assumption violations and quantify their magnitude. Such effect sizes are not currently commonly used for the two main assumptions that were discussed so far in the paper (normality and constant variance), but can be used for other important assumptions.

A key example of this appears in the context of Poisson regression models, a type of *generalized linear model* (GLM), used for working with count data (Forthmann & Doebler, 2021; Green, 2021; Winter, 2019).^7^ The Poisson distribution assumes that the mean and the variance of the data are equal (i.e., that there is *equidispersion*), but this assumption is often violated (Coxe et al., 2009). This can be either due to *overdispersion*, when the variance is bigger than expected, or *underdispersion*, when the variance is smaller than expected (Brooks et al., 2017; Forthmann & Doebler, 2021). Overdispersion leads to underestimated (i.e., *liberal*) standard errors, *p*-values, and confidence intervals, while underdispersion leads to overestimated (i.e., *conservative*) standard errors, *p*-values, and confidence intervals (Brooks et al., 2017; Forthmann & Doebler, 2021). Accordingly, it is important to check dispersion when using Poisson models, and based on the results of these checks, researchers may, for example, choose to use alternative methods, like negative binomial models (Brooks et al., 2017, 2019; Winter, 2019).

One way to check dispersion is to use the *testDispersion* function in the *DHARMa* package in R (Hartig, 2021), which compares the variance of a model’s observed residuals against the variance of its expected residuals (as determined based on simulations).^8^ This outputs a *p*-value for the dispersion test, together with a dispersion ratio as an effect size, where a ratio > 1 indicates overdispersion, while a ratio < 1 indicates underdispersion.

In a large-scale simulation (*N* = 150,000) with a Poisson model, this function outputted the following results: *ratio*_*dispersion*_ = 0.98, *p* < .001. Relying only on the *p*-value would suggest that there is a statistically significant deviation from the expected dispersion, but will not reveal whether the problem is overdispersion or underdispersion, or what is the magnitude of the deviation from the expected dispersion. However, looking at the associated effect size (the dispersion ratio) reveals that there is underdispersion (since the ratio < 1), and more importantly, that this deviation from the expected dispersion is very small (since the dispersion ratio is very close to 1) (Hartig, 2021).

A related issue with Poisson models is *zero-inflation*, which occurs when count data contains more zeros than expected (Brooks et al., 2017, 2019). This issue can cause biased parameter estimates, and can be addressed using solutions like zero-inflated models (Brooks et al., 2017, 2019; Green, 2021; Harrison, 2014).

One way to check for this issue is to use the *testZeroInflation* function in the DHARMa package, which is similar to the *testDispersion* function (Hartig, 2021). This function compares the observed number of zeros with expected number of zeros (based on simulations). It outputs a *p*-value for the associated test, together with a ratio of observed to expected zeros as an effect size, where a ratio < 1 indicates that the observed data has fewer zeros than expected, while a ratio > 1 indicates that it has more zeros than expected (i.e., has zero-inflation).

In a large-scale simulation (*N* = 150,000) with a Poisson model, this function outputted the following results: *ratio*_*observed_to_expected_zeros*_ = 1.01, *p* < .001. Again, relying only on the *p*-value would suggest that there is a statistically significant deviation from the expected number of zeros. However, this will not reveal whether there are more or fewer zeros than expected (despite the name of this function, it tests both possibilities by default), or how big the deviation is. Looking at the associated effect size (the ratio of observed to expected zeros) reveals that there are more zeros than expected in the model (since the ratio > 1), but that this deviation from the expected ratio is again very small (since the ratio is very close to 1) (Hartig, 2021).

**Fig. 9:**
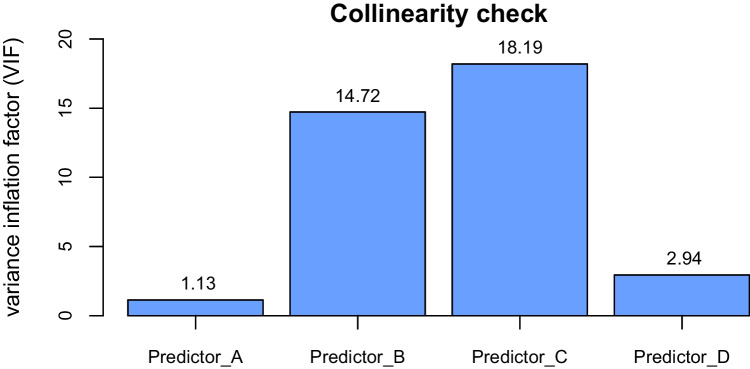
An example collinearity check for a multiple regression model, where a higher VIF indicates greater collinearity

Finally, another example of a numerical effect size that can be used in statistical diagnostics is the *variance inflation factor* (VIF). It quantifies the severity of *collinearity* (or *multicollinearity*) in regression models, where a higher VIF indicates greater collinearity, and consequently greater inflation in the standard errors of the coefficients (Alin, 2010; Dormann et al., 2013). VIF is often shown using plots like the one in Fig. 9, which provide a visual representation of this numeric measure.

### Remember that assumption tests can be useful

As we saw previously, assumption tests have certain limitations, such as that they do not provide information about the way in which observed distributions differ from expected. Nevertheless, assumption tests can sometimes be useful, primarily when they complement other types of assumption checks (Flatt & Jacobs, 2019; Hartig, 2021).

A key example of this appears in statistical diagnostics of Poisson models, which, as discussed in the previous sub-section, should generally be checked for overdispersion and underdispersion by calculating the relevant effect size (dispersion ratio) (Hartig, 2021). When doing this, it can sometimes be beneficial to run an associated significance test for the difference between the observed and expected dispersion—while considering the sample’s size—as this can help assess whether a certain deviation from expected might be due to chance (Hartig, 2021).

In addition, assumption tests can also be beneficial when used for initial diagnostics. For example, tests can be used in this manner when generating a large number of models (e.g., hundreds or thousands), which may be infeasible to assess visually; in such situations, the assumption tests may be used to identify a subset of models that are more likely to have issues, and these models can then be inspected visually. As with the assessment of dispersion, here too it is possible to use further statistics when assessing the results from the assumption tests, and particularly effect size (where available) and sample size.

When using assumption tests in this and other capacities, it is important to remember the issues that they can involve (e.g., false negatives), and to make sure that you are minimizing the risks of those issues (e.g., by considering whether your samples are large enough for tests to detect non-normality). This also involves considering the assumptions that these tests have, and choosing tests that are most appropriate for your situation.

In addition, when deciding whether and how to use assumption tests, you should consider other relevant factors, including how concerned you are over false positives/negatives, and how you want to balance the validity of your analyses with the time spent checking their assumptions. For example, if you need to run diagnostics on a large number of models (e.g., 100) for a preliminary analysis where you are not concerned about false positive/negatives, then you might decide that for your specific purposes it is better to use automated assumption tests rather than visual checks. Alternatively, if you are conducting diagnostics and are more concerned over false negatives than false positives (e.g., since you can visually assess any models of concern), then you may change your *p*-value threshold to reflect this (e.g., from .05 to .10), while again also considering factors such as the size of your samples (Bishara et al., 2021).

Finally, note that it may be beneficial to use *equivalence tests* in this context, rather than traditional NHST. Such tests “examine whether the hypothesis that there are effects extreme enough to be considered meaningful can be rejected” (Lakens et al., 2018, p. 260). They add flexibility to the diagnostic process, by enabling researchers to set the quantitative bounds involved in rejecting the hypothesis, based on relevant evidence. In the context of normality, for example, using these tests can lead people to move from asking “can we reject the hypothesis that the data are normally distributed?”, to asking “is this distribution consistent enough with a normal distribution for our present purposes?”

### Remember that visualizations and effect sizes are imperfect

Despite the potential benefits of using visual checks and effect sizes in statistical diagnostics, it is important to remember that they have limitations.

One limitation of visual checks is that they may not detect certain issues. For example, the panel of assumption checks for linear models that was shown in Fig. 8 is not expected to detect dependence between observations (e.g., due to the presence of multiple data points per participant). Furthermore, people sometimes fail to notice issues that are evident in visualizations (e.g., non-normality), due to errors in judgment (Bishara et al., 2021; Fisch, 1998). This issue may be worse in small samples (e.g., just 10 observations), where it can be harder to interpret visual patterns with confidence (Cook & Weisberg, 1983; Weissgerber et al., 2016). The opposite issue can also occur, when people *overinterpret* visual patterns. For example, this can involve believing that a certain residual pattern is indicative of substantial non-normality, when in reality it is merely the result of some trivial noise. Psychologically, the tendency to see such patterns in random noise can be considered a form of *apophenia* (Wickham et al., 2010), and can be attributed to causes like Gestalt principles (Dixon, 2012).

Another limitation of visual checks is that they can have misleading results in some cases. For example, as Cook and Weisberg (1983) note, using residual plots to check for constant variance can wrongly seem to indicate that there is non-constant variance if the density of the points is uneven along the *x*-axis, since areas with higher density generally also have a greater spread on the *y*-axis, due to the increased number of observations.

Another issue with visual checks is the subjectivity involved in the interpretation of visual patterns, which can cause issues like biased interpretation of results (Bishara et al., 2021). Furthermore, there is often arbitrariness in the choice of which graphical methods to use. For example, visual normality checks can be performed using many methods other than the density plots that were used in this paper, including *histograms*, *boxplots* (also called *box-and-whisker plots*), and *normal quartile plots* (also called *Q-Q plots*) (Das & Imon, 2016; Mishra et al., 2019; Pole & Bondy, 2012). Moreover, many of these methods have various settings that can influence their interpretation, like bin size in histograms and the opacity of points in dot plots (Correll et al., 2019). Similarly to the choice of which assumption tests to use, the choice of which graphical methods to use increases the researcher degrees of freedom (Gelman & Loken, 2014; Simmons et al., 2011; Wicherts et al., 2016). This, in turn, can lead to issues like selecting a method because it supports one’s hypotheses rather than because it is the most appropriate method to use, for instance due to an unconscious confirmation bias (Veldkamp, 2017; Wicherts et al., 2016).

Finally, visual checks may also be infeasible in some cases. For example, this can be the case if you need to deal with a very large number of models (e.g., 5000), which may take too long for you to assess visually.

Effect sizes also have various limitations when used in statistical diagnostics. For example, consider the VIF, which was mentioned previously as a measure of effect size for collinearity. One issue with VIF is that it is often interpreted using arbitrary rules of thumb, for instance when a VIF of 4 or 10 is considered to indicate the presence of “severe” collinearity, which merits a change to analyses (O’Brien, 2007). This can lead to a similar issue with false binarity as the *p* threshold of .05 (as discussed in §3.2), for instance if a VIF of 4.01 is viewed as indicating severe collinearity, while a VIF of 3.99 does not.

Furthermore, VIF values are often considered in isolation, based only on their magnitude, but other factors, like sample size, also play a key role in determining how substantial the impact of collinearity is on associated statistical inferences (O’Brien, 2007).^9^ In addition, the influence of collinearity should be considered in the context of the inferential goals of the analysis. As Belsley et al. (2004, p. 116) note:

… for example, if an investigator is only interested in whether a given coefficient is significantly positive, and is able, even in the presence of collinearity, to accept that hypothesis on the basis of the relevant t-test, then collinearity has caused no problem. Of course, the resulting forecasts or point estimates may have wider confidence intervals than would be needed to satisfy a more ambitious researcher, but for the limited purpose of the test of significance [initially] proposed, collinearity has caused no practical harm… These cases serve to exemplify the pleasantly pragmatic philosophy that collinearity doesn’t hurt so long as it doesn’t bite.

Finally, another issue with VIF is that it merely quantifies the degree of collinearity present in the data, but does not say anything about what type of collinearity exists (Alin, 2010), which is reminiscent of the issue of limited descriptiveness of assumption tests discussed in §3.3. This is problematic, since different types of collinearity may necessitate different responses. For example, Iacobucci et al. (2016) show that using mean centering reduces what they refer to as “micro” collinearity, but not “macro” collinearity.

All this does *not* mean that visual checks and effect sizes should be avoided in statistical diagnostics, but rather that they should be used with appropriate caution, similarly to other statistical methods, like assumption tests.

### Use proper terminology

Because of the issues associated with assumption testing, it is important to draw a clear terminological distinction between *testing* and *checking* assumptions. Specifically, the term “assumption testing” should only be used to refer to the testing of assumptions using statistical tests (e.g., the Shapiro–Wilk test). Conversely, the term “assumption checking” can be used to refer to all forms of assumption checks, including statistical tests, as well as visualizations and numerical assessments of effect sizes.

In addition to enabling a more nuanced discussion of statistical diagnostics, drawing this distinction will help emphasize the importance of considering more than just statistical significance when checking assumptions. Doing this may, in turn, help prevent certain cases where people are told to “test” their assumptions, and interpret this as meaning that they should only use statistical tests in their diagnostics. This is particularly relevant for researchers with a limited statistical background, who are less likely to understand the issues with assumption tests, and who comprise a substantial portion of those who use statistical methods in practice (Hu & Plonsky, 2021).

### Key practical recommendations

Based on the material discussed in the paper so far, the following are key practical recommendations for conducting statistical diagnostics:

1. Remember the potential issues with assumption checks when deciding whether/how to use them, and when interpreting their results.
2. Prefer the most appropriate type of assumption check to use in your particular situation (e.g., visualization over a test), even if other methods are more common.
3. Use a combination of diagnostic methods where appropriate (e.g., significance testing initially, followed by visualization).
4. Draw a terminological distinction between assumption *testing* (which involves only statistical tests) and assumption *checking* (which can involve statistical tests, as well as other methods, including visualization and numerical effect sizes).
5. Prefer using existing programmatic tools for diagnostics, and using their default settings, unless you have a compelling reason to do otherwise. This can make the diagnostic process easier to implement, more replicable, and more comparable across studies, while also reducing researcher degrees of freedom (Gelman & Loken, 2014; Simmons et al., 2011; Wicherts et al., 2016). Examples of relevant tools include the *performance* and *DHARMa* R packages, whose use was demonstrated earlier.
6. Explain the rationale behind your diagnostic process, including what you checked, how, and why, and how you interpreted the results. When doing this, acknowledge any important limitations and arbitrariness in your process, for example if there were other reasonable methods you could have used.
7. Share all the material that you used in the diagnostics, like the code that you used and the resulting plots. It may be best to do this as part of online supplementary material, particularly if space constraints would otherwise prohibit you from sharing important information (Hu & Plonsky, 2021).
8. Judge assumption violations as a complex spectrum, rather than a simplistic binary. This means that you should consider not only whether there is a violation, but also what the violation is, what caused it, how severe it is, and how it affects your particular analyses, while also considering factors like the robustness of your methods to this type of violation, the size of your sample, and your inferential goals. You may realize that your method is robust enough or the violation is minor enough that nothing needs to be done, especially when analyzing large samples, which are usually—but not always—more robust to violations (Casson & Farmer, 2014; Ernst & Albers, 2017; Fagerland, 2012; Ghasemi & Zahediasl, 2012; Knief & Forstmeier, 2021; Kozak & Piepho, 2018; Lumley et al., 2002; Pole & Bondy, 2012; Poncet et al., 2016; Schmidt & Finan, 2018; Tijmstra, 2018). This is often the case, for example, with violations of normality (Knief & Forstmeier, 2021) or collinearity (O’Brien, 2007). You may also realize that even if the violation is substantial, it does not affect the goals of your particular analyses. For example, if the goal of an analysis is mainly inference of the regression line, rather than prediction of individual data points, then the normality assumption might not be important (Gelman et al., 2022).
9. Remember that statistical diagnostics cannot detect all the potential issues in statistical methods. For example, such diagnostics cannot, in many cases, detect key issues with the validity of the model specification (e.g., omitted-variable bias) or with the representativeness of the sample (e.g., systematic biases in the sampling process). However, these issues can be far more important than issues with normality and constant variance (Gelman et al., 2022), as mentioned in Appendix 1. Accordingly, it is important to consider such issues before running your analyses, even if you cannot check them using formal methods. Another example of such an issue are inappropriate causal interpretations of regression results, which might only be identified by doing things like assessing the language used to present results (Bordacconi & Larsen, 2014).
10. Remember that assumption checks cannot guarantee that your analyses are correct. Rather, they provide evidence—which you then assess—regarding the presence and severity of certain assumption violations. As such, they can only increase your confidence that your analyses are not grossly wrong, or provide you with information regarding what kind of issues they suffer from (Faraway, 2016; Hartig, 2021; Winter, 2019). In this regard, it helps to remember Box’s aphorism that “All models are wrong but some are useful” (Box, 1979﻿, p. 202), and his recommendation to worry selectively: “Since all models are wrong the scientist must be alert to what is importantly wrong” (Box, 1976, p. 972).

## Conclusions

When using statistical methods like linear models, you should generally check if and how their assumptions are violated. This is because assumption violations can have various consequences, so assessing assumptions is crucial to deciding whether and how to proceed with analyses. A common way to do this is to use statistical tests, like the Shapiro–Wilk test of normality, but as shown in the present paper, this approach involves various potential issues, including statistical errors (false positives and false negatives), false binarity, limited descriptiveness, misinterpretation (e.g., of *p*-values as effect sizes), and potential testing failure due to unmet test assumptions.

Despite this, assumption tests can sometimes be beneficial. However, the aforementioned issues mean that if assumption tests are used, then this should be done with caution, and generally to supplement visualization and/or numerical effect sizes, though these types of assumption checks also have limitations. In addition, assumption checks can also be improved by following other practical guidelines which were outlined in the paper, including explaining the rationale behind the diagnostic process, sharing all the relevant material, and judging assumption violations as a complex spectrum.

## Figures and Tables

**Fig. 10:**
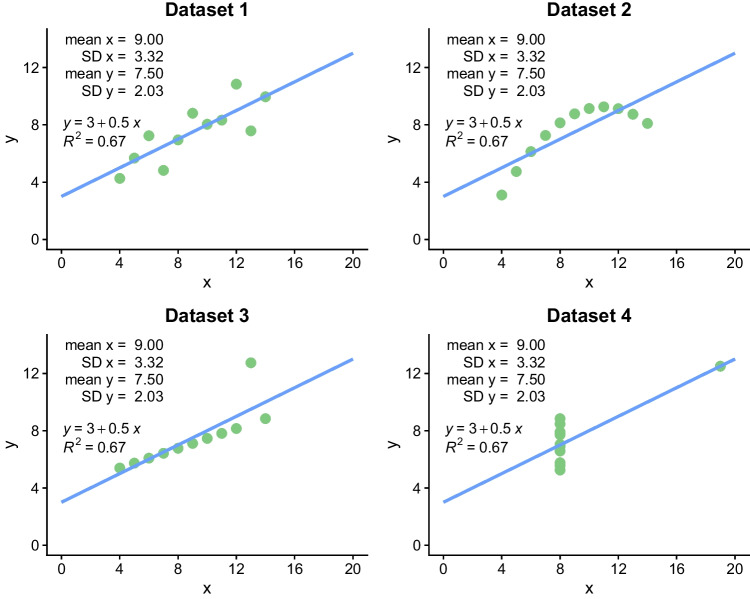
Plots of *Anscombe's quartet* (Anscombe, 1973). The observations (green points) are distributed very differently across each dataset. However, despite this, the mean and SD of each variable are the same across the datasets, as are the regression equations (represented by the blue line) and corresponding *R﻿*^2^

**Fig. 11:**
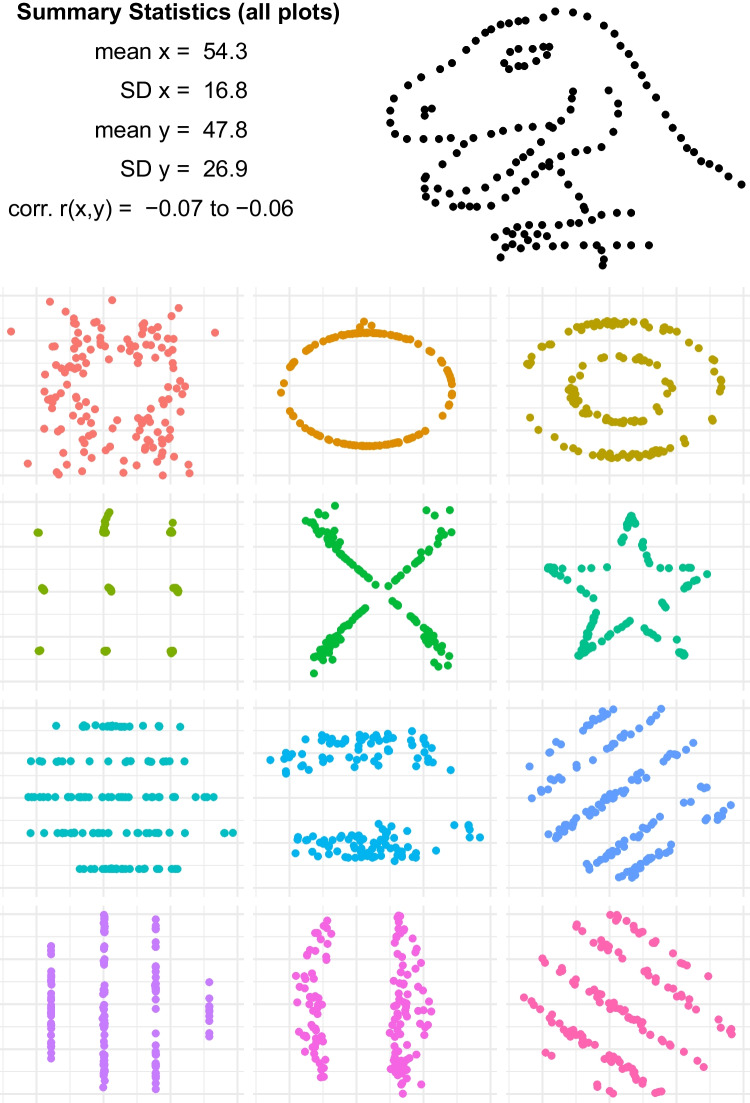
The *Datasaurus dozen* (Matejka & Fitzmaurice, 2017). The datasets in all the plots—including the dinosaur—have functionally identical summary statistics.

## Appendices

### Appendix 1: Assumptions of linear regression models

The following is a brief summary of the key assumptions of simple linear regression models. It is based primarily on the description of Gelman et al. (2022, pp. 153–155), who also ranked the assumptions in the following (decreasing) order of importance:

1. ***Validity*****.** The data should map to your research question, by (i) having an outcome that accurately reflects the phenomenon of interest, (ii) including all relevant predictors in the model, and (iii) the model generalizing to all cases to which it will be applied.
2. ***Representativeness*****.** The sample should be representative of its parent population, primarily in terms of the distribution of the response variable given the predictors in the model.
3. ***Additivity and linearity*****.** The deterministic component of the model should be a linear function of its separate predictors. Addressing violations of this can involve things like adding interactions or transforming the data.
4. ***Independence of errors*****.** The model’s errors should be independent of one another. Addressing violations of this can involve things like using mixed-effects models. Note that this and the other assumptions involving errors are checked by examining the model’s *residuals*, which approximate the errors, since they cannot be observed directly (Cook & Weisberg, 1999; Knief & Forstmeier, 2021).
5. ***Equal variance of errors*****.** The variance in the errors should be constant across all levels of the independent variables (i.e., along the regression line) (Barker & Shaw, 2015; Winter, 2019). This means that the conditional variance of the response variable is the same for all observations (Cribari-Neto & Zarkos, 1999; Fox, 2022). This assumption is also called *constant variance*, *homogeneity of variance*, and *homoscedasticity*. It is contrasted with *unequal variance*, which is also called *non-constant variance*, *heterogeneity of variance*, and *heteroscedasticity*.
6. ***Normality of errors*****.** The errors should be normally distributed. Note that this applies only to the errors, so while the conditional distribution of the response as a function of the predictors should be normal, the raw response and predictors can be non-normal.

An important caveat is that there is variability in the literature in terms of which assumptions are discussed and how, due to things like varied conventions across fields. For example, a list of regression assumptions in Fox (2022) explicitly mentions most of the mathematical assumptions listed above (linearity, constant variance, normality, and independence), but not additivity, and not the conceptual assumptions (validity and representativeness). Alternatively, in econometrics, the focus and framing of the discussion often revolves around the *Gauss-Markov assumptions*, and includes concepts like exogeneity, auto-correlation, and omitted-variable bias (Belsley et al., 2004; Verbeek, 2008).

In addition, there are two other important caveats about these assumptions. First, certain issues that are important to consider in statistical diagnostics are not always considered assumptions. For example, while Fox (2022) mentions lack of (perfect) collinearity as an assumption, Gelman et al. do not, although they do discuss collinearity as a potential issue elsewhere in their text. Similarly, although Fox does not mention lack of outliers as an assumption, he does discuss outliers as a potential issue elsewhere in his text. The second caveat is that other types of models can have other assumptions, like the *equidispersion* assumption of Poisson GLMs, which was discussed in the paper (Coxe et al., 2009).

### Appendix 2: Informativeness of graphical methods

In addition to the simulations that were shown in the body of the paper, another way to illustrate the potential benefits visualization in statistical analyses is through datasets that demonstrate how data with very different distributions can have the same summary statistics. Included below are two such datasets— *Anscombe's quartet* and the *Datasaurus dozen*—which are often used for this purpose.

#### Anscombe's quartet

Appendix Fig. 10 contains plots of *Anscombe's quartet* (Anscombe, 1973), showing four datasets with very different distributions of observations, but functionally identical summary statistics. These datasets demonstrate how visualization can be useful for detecting many common types of assumption violations (Cook & Weisberg, 1999; Hox et al., 2018; Winter, 2019); for example, dataset 2 reveals a pattern of non-linearity.

Data for the plots came from the *anscombe* dataset in base R (R Core Team, 2021), which is based on Anscombe (1973).

#### Datasaurus dozen

Appendix Fig. 11 contains plots of the *Datasaurus dozen* (Matejka & Fitzmaurice, 2017), showing 13 (i.e., a baker’s dozen) datasets with very different distributions of observations but functionally the same summary statistics.

Data ﻿for the plots came from the *datasauRus* package in R (Davies et al., 2022), which is based on the datasets from Matejka and Fitzmaurice (2017) and the original Datasaurus by Cairo (2016).

## Footnotes

1 This assumption actually pertains to the models’ *errors*, which cannot be directly observed, and which are therefore estimated using the residuals (Barker & Shaw, 2015; Bilon, 2021; Cook & Weisberg, 1999; Knief & Forstmeier, 2021; Pek et al., 2018). Accordingly, throughout the paper, we will discuss this and related assumptions in the context of residuals, rather than errors. However, this approach has some flaws, like the problem of *supernormality*, where residuals appear to be normal even if the errors are not normal, particularly in small samples (Gnanadesikan, 1997; Weisberg, 2005). Such issues led to the development of various types of residuals, like *recursive residuals*, which may sometimes be better suited for diagnostic purposes than raw residuals (Hawkins, 1991; Kianifard & Swallow, 1996).

2 It is unclear to what extent the issue is that assumptions are not checked, as opposed to being checked but not reported (Hu & Plonsky, 2021). Nevertheless, the low rates of reporting—and especially of *correct* reporting—suggest there is a lack of understanding of the importance of assumption checking, and of how to do it properly (Hoekstra et al., 2012; Hu & Plonsky, 2021; Nielsen et al., 2019; Nimon, 2012). In addition, even when assumptions are checked correctly, a lack transparency regarding the checks (i.e., due to lack of reporting) can cause issues for researchers who try to interpret, compare, or replicate a study's analyses (Ernst & Albers, 2017; Hu & Plonsky, 2021).

3 However, it does not eliminate these issues entirely. For example, researchers may need to choose between multiple available tests, such as the Shapiro–Wilk test and the *Kolmogorov-Smirnov (K-S) test*, where the K-S test generally has lower power to detect non-normality (Ghasemi & Zahediasl, 2012; Steinskog et al., 2007). Furthermore, researchers also be faced with many other alternatives to choose from, which can also lead to different results, such as the *Lilliefors test*, the *Anderson–Darling test*, and the *Jarque-Bera test* (Das & Imon, 2016; Ghasemi & Zahediasl, 2012; Pole & Bondy, 2012; Steinskog et al., 2007).

4 Note that a “strong” violation (e.g., substantial deviation from normality) does *not* necessarily entail “strong” consequences for analysis. For example, in a simulation study, Knief and Forstmeier (2021) found that “Gaussian models are robust to non-normality over a wide range of conditions, meaning that *p*-values remain fairly reliable except for data with influential outliers judged at strict alpha levels. Gaussian models also performed well in terms of power across all simulated scenarios. Parameter estimates were mostly unbiased and precise except if sample sizes were small or the distribution of the predictor was highly skewed.” (p. 2576).

5 As Rosnow & Rosenthal (1989, p. 1277) state: “…surely, God loves the .06 nearly as much as the .05. Can there be any doubt that God views the strength of evidence for or against the null as a fairly continuous function of the magnitude of *p*?” This reflects the continuous—rather than binary—nature of *p*-values, as well as the fact that although the associated threshold of .05 is widely adopted, it is also arbitrary (Gelman & Stern, 2006; Greenland et al., 2016). Essentially, this issue means that a tiny quantitative difference in *p*-value leads to a disproportionate qualitative difference in analyses, as going from *p* = .051 to *p* = .049 means that the distribution is suddenly considered “non-normal.”

6 The ease of use of these *turnkey* tools—where you only need to provide the data and potentially choose some basic settings—might help increase the rate of assumption checking over time.

7 Count data can be equal only to zero or positive integers (i.e., it can take values like 0, 1, 2, 3…) (Green, 2021; Winter, 2019). It is used to model things like the number of speech errors in a text (Winter, 2019), the number of publications of a researcher (Forthmann & Doebler, 2021), or the number of times someone exercises per week (Green, 2021).

8 For more information on this approach to diagnostics of GLMs and GLMMs (*generalized linear mixed models*), see the DHARMa documentation (Hartig, 2021). The documentation also demonstrates how visualizations can complement the dispersion test. One example of this is by showing a histogram of expected variance values based on simulations, which can help to see how different the observed variance is from the expected. Another example of this is by showing a QQ plot of the residuals, which can help to visually assess dispersion and other potential issues (like deviation from normality). The DHARMa documentation also shows how these diagnostics can be generated through a few simple functions in R, in a similar way to how checks for LMs can be generated using the *performance* package, as was shown in the previous sub-section.

9 As O’Brien (2007, p. 675) notes: “…unless the collinearity is perfect, increasing the sample size (using more cases of the same sort) will reduce the variance of the regression coefficients” and “When the focus is on the variance of a regression coefficient and the stability of result: the sample size, the proportion of the variance in the dependent variable associated with the independent variables, the variance of the independent variable whose coefficient is of concern, and the multi-collinearity of the independent variable of concern with the other independent variables are all important.”
